# Supplementary material for: Predicting visual function by interpreting a neuronal wiring diagram
Source: Nature. 2024 Oct 2;634(8032):113–23. doi: 10.1038/s41586-024-07953-5 (PMC11446822; doi:10.1038/s41586-024-07953-5)
Supplement: Supplementary file 6 — CRF and ERF predictions for individual TmY4 and TmY9 cells. Analogous to Supplementary Data 3, but for TmY target types. Shown are the top four monosynaptic pathways, the strongest pathway passing through each of the top ten intermediary types (ranking from Extended Data Fig. 7), and the trisynaptic pathway Tm1–TmY–Dm3–TmY (see the section entitled Prediction of spatial normalization). [file 41586_2024_7953_MOESM6_ESM.zip › DataS4/TmY9qΓèÑ/720575940617536473.pdf]

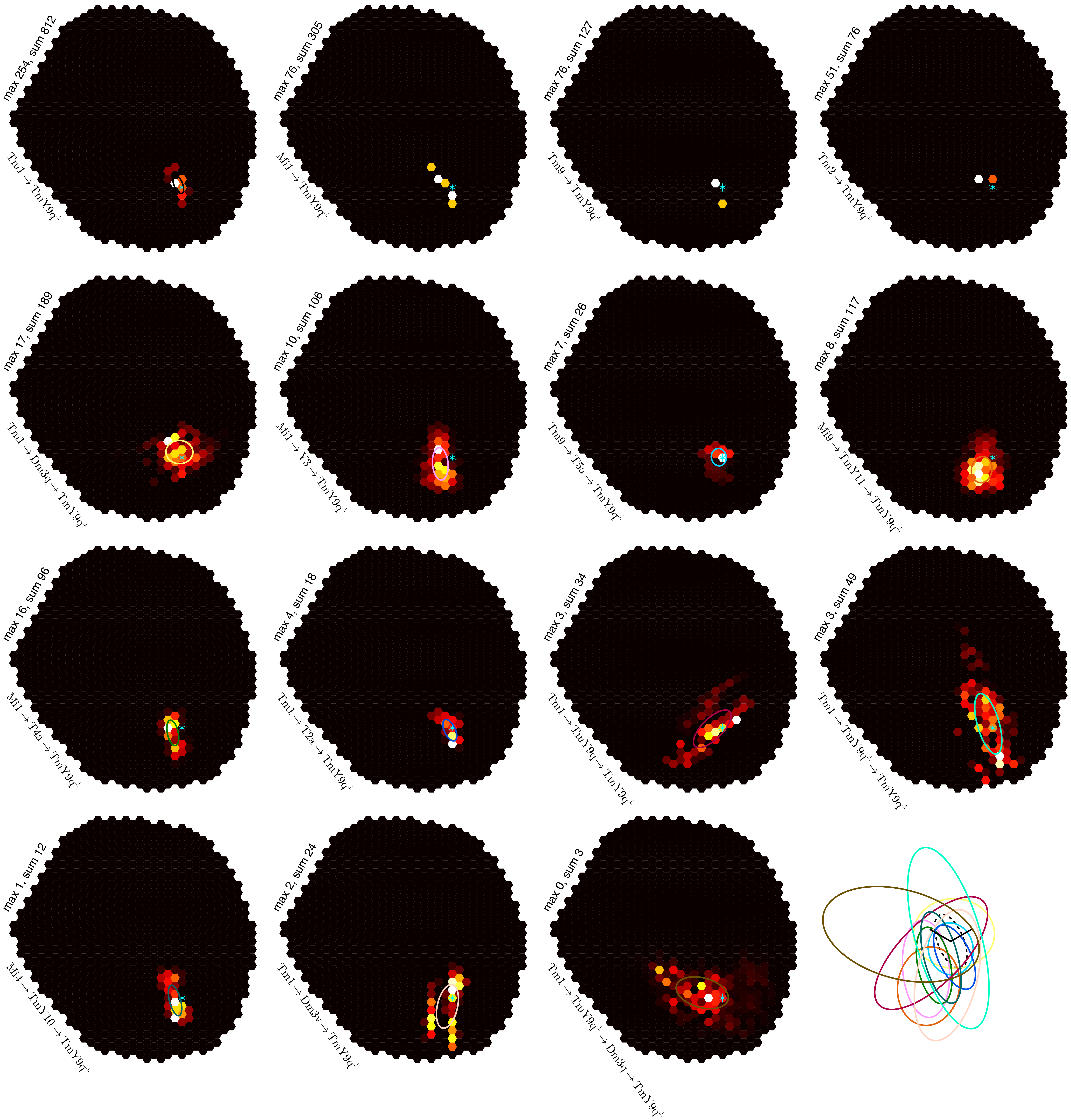
max 254, sum 812
Tm1 → TmY9q⊥
max 76, sum 305
Mi1 → TmY9q⊥
max 76, sum 127
Tm9 → TmY9q⊥
max 51, sum 76
Tm2 → TmY9q⊥
max 17, sum 189
Tm1 → Dm3q → TmY9q⊥
max 10, sum 106
Mi1 → Y3 → TmY9q⊥
max 7, sum 26
Tm9 → T5a → TmY9q⊥
max 8, sum 117
Mi9 → TmY11 → TmY9q⊥
max 16, sum 96
Mi1 → T4a → TmY9q⊥
max 4, sum 18
Tm1 → T2a → TmY9q⊥
max 3, sum 34
Tm1 → TmY9q → TmY9q⊥
max 3, sum 49
Tm1 → TmY9q⊥ → TmY9q⊥
max 1, sum 12
Mi4 → TmY10 → TmY9q⊥
max 2, sum 24
Tm1 → Dm3v → TmY9q⊥
max 0, sum 3
Tm1 → TmY9q⊥ → Dm3q → TmY9q⊥
